# The Epidemiology of Zoonotic Brucellosis in Bahr el Ghazal Region of South Sudan

**DOI:** 10.3389/fpubh.2019.00156

**Published:** 2019-06-26

**Authors:** Nuol Aywel Madut, James Muleme, Clovice Kankya, George William Nasinyama, John Bwalya Muma, Jacques Godfroid, Ambrose Samuel Jubara, Adrian Muwonge

**Affiliations:** ^1^Department of Clinical Studies, Faculty of Veterinary Science, University of Bahr el Ghazal, Wau, South Sudan; ^2^Department of Biosecurity, Ecosystems & Veterinary Public Health (BEP), College of Veterinary Medicine Animal Resources & Biosecurity (COVAB), Makerere University, Kampala, Uganda; ^3^Department of Disease Control and Environmental Health, College of Health Sciences, Makerere University, Kampala, Uganda; ^4^Department of Disease Control, School of Veterinary Medicine, University of Zambia, Lusaka, Zambia; ^5^Department of Arctic and Marine Biology, Faculty of Biosciences, Fisheries and Economics, University of Tromsø - The Arctic University of Norway, Tromsø, Norway; ^6^Division of Genetics and Genomics, The Roslin Institute, University of Edinburgh, Edinburgh, United Kingdom

**Keywords:** human, brucellosis, cattle, risk groups, epidemiology, South Sudan

## Abstract

**Background:** In this study, we focused on three zoonotic brucellosis risk groups; abattoir workers, febrile cases at Wau hospital and cattle herders, in Bahr el Ghazal region, South Sudan. Competitive c-ELISA was used to detect anti-*Brucella* antibodies in 725 individuals between December 2015 and May 2016. In addition, questionnaire metadata, focus group discussions and key informant interviews were used to characterize the epidemiology of zoonotic brucellosis in this region.

**Results:** Overall, we estimate 27.2 % (95% CI = 23.9–30.6) brucellosis sero-prevalence; 32.1% (95% CI = 26.2–38.4), 23.0% (95% CI = 19.1–27.4) and 34.6% (95% CI = 24.4–46.3) among abattoir workers, febrile cases, and herders, respectively. Marital status (Single, OR = 0.58, 95%CI: 0.36–0.91, *P* = 0.02) and ethnicity (Kerash OR = 6.01, 95%CI: 1.97–21.10, *P* = 0.003 and Balanda, OR = 3.78, 95%CI: 1.42–12.02, *P* = 0.01) were associated with brucellosis. While gender and ethnicity were important factors for general awareness of zoonotic diseases. Highly ranked occupations at risk included veterinarian, butchers and milk handlers. We also identified covariate patterns for clinical diagnostics and public health interventions.

**Conclusion:** We report the highest sero-prevalence of zoonotic brucellosis in three risk groups in the East African region. All this is not only occurring in a population with limited awareness that brucellosis is a zoonotic disease but also where one in nine health workers tested was sero-positive. We identified social demographic associations with brucellosis, however, the qualitative analysis suggests these are more complex and nuanced. Therefore, future studies could benefit from the use of the mixed methods approach to add extensiveness and depth to our understanding of zoonotic disease drivers, in order to implement mitigating measures such as cattle vaccination.

## Introduction

Brucellosis is a neglected bacterial zoonotic disease that is transmitted through consumption of unpasteurized milk, undercooked meat from infected animals, or by contact with their secretions (1). The disease came to prominence during the 1850's Crimean war in Malta, which claimed thousands of British soldiers, hence the name “Malta disease” (2). Unlike tuberculosis, the case fatality due to brucellosis is extremely low, and the morbidity [expressed in disability-adjusted life years (DALYs)] is complex to estimate (3), hence the lack of annual epidemiological reporting at global level (4, 5). The disease is caused by four species of *Brucella*; *B. melitensis* (mainly goats, sheep, camels, but also cattle), *B. suis* (pigs), *B. abortus* (mainly cows, buffalo, camels, yaks, but also sheep and goats), and *B. canis* (dogs). All the four species can cause disease in human however, *B. melitensis* is the most prevalent (6). The disease is characterized by a wide range of signs in humans, however, undulating febrile episodes, night sweats and muscular weakness ultimately leading to abortion in pregnant women are the most noticeable signs (7, 8). Brucellosis has been eradicated in some developed countries, it however remains to be seen if the strategies currently implemented in Latin America and the Mediterranean countries will yield similar results (9). In Sub Saharan Africa (SAA), brucellosis is endemic in animals (10, 11) and because of the weak food safety and public health infrastructure, it is arguably endemic in the human population as well. The epidemiological overlap of brucellosis and other febrile-centric diseases in humans like malaria, makes differential diagnosis in areas with weak diagnostic capacity extremely complicated and expensive (12, 13). This is possibly why clinicians in SAA attribute most febrile cases to malaria, even though approximately 50-80% of fevers result from other causes (13) like brucellosis, Rift Valley Fever, bird flu and Yellow Fever among others (14). The clinical and epidemiological picture is further compounded by the disproportionately low levels of resourcing for disease management, surveillance and strategic control (13, 15). Diseases like brucellosis that are primarily augmented by the host environment have the potential to cripple entire human communities, and yet the strategic control approaches rest on the rarely documented dynamics of such communities (16). Some countries like Ethiopia have instituted *ad-hoc* multidisciplinary task forces under the one-health umbrella to spearhead community centered zoonotic disease control. Such efforts aim at documenting disease dynamics to support context-specific policy formulation (17). A country like South Sudan currently lacks both the evidence-based strategy and the necessary peaceful environment to implement any zoonotic disease control programs (18, 19). This not only represents a genuine trans-boundary disease control challenge for the region but also for the South Sudanese communities, for example; the absence of a livestock and livestock products inspection system means that infected animals can be imported, reared and slaughtered for human consumption (20). Like many other SAA countries, the risk of brucellosis varies along the livestock and foods of animal origin value chain. The high-risk groups will be people that interact with live livestock, slaughtered livestock, and or milk and its products. Such groups include herders, dairy processors, veterinarians, butchers and the general public that consumes products of livestock origin (21, 22). There is a specific absence of epidemiological data on brucellosis for these high-risk groups in South Sudan, possibly because the country has been at war for the last half of a century (23). Such information is critical for designing robust and sustainable control measures to support the growing health care system in South Sudan. For example; by identifying the species of *Brucella*, hotspots for livestock reservoir species, risk groups and drivers can be mapped for national task groups to design and implement cost effective targeted public health interventions (24). Recently, in Sudan, *Brucella melitensis* and the S19 anti-*Brucella abortus* vaccine strain were isolated from farmworkers employed at two cattle farms (25), while *Brucella abortus* has been isolated from sheep in the Kassala state Eastern Sudan (26).

These examples highlight the complexity and need to understand brucellosis at the livestock-human interface where different *Brucella* species exist and mixed herding practiced (27). Therefore, we focused on three risk groups; cattle herders, abattoir workers and febrile cases at Wau hospital in order to unpick these complex dynamics in Bahr el Ghazal region of South Sudan. We screened these individuals for *Brucella* antibodies, examined their awareness of zoonotic diseases, as well as profiled the socio-anthropological factors associated with being sero-positive. Furthermore, we used the dataset generated to identify covariate patterns that could be used to enhance clinical diagnostics as well as contextualize public health intervention in South Sudan.

## Materials and Methods

### Study Design

**Figure 1:**
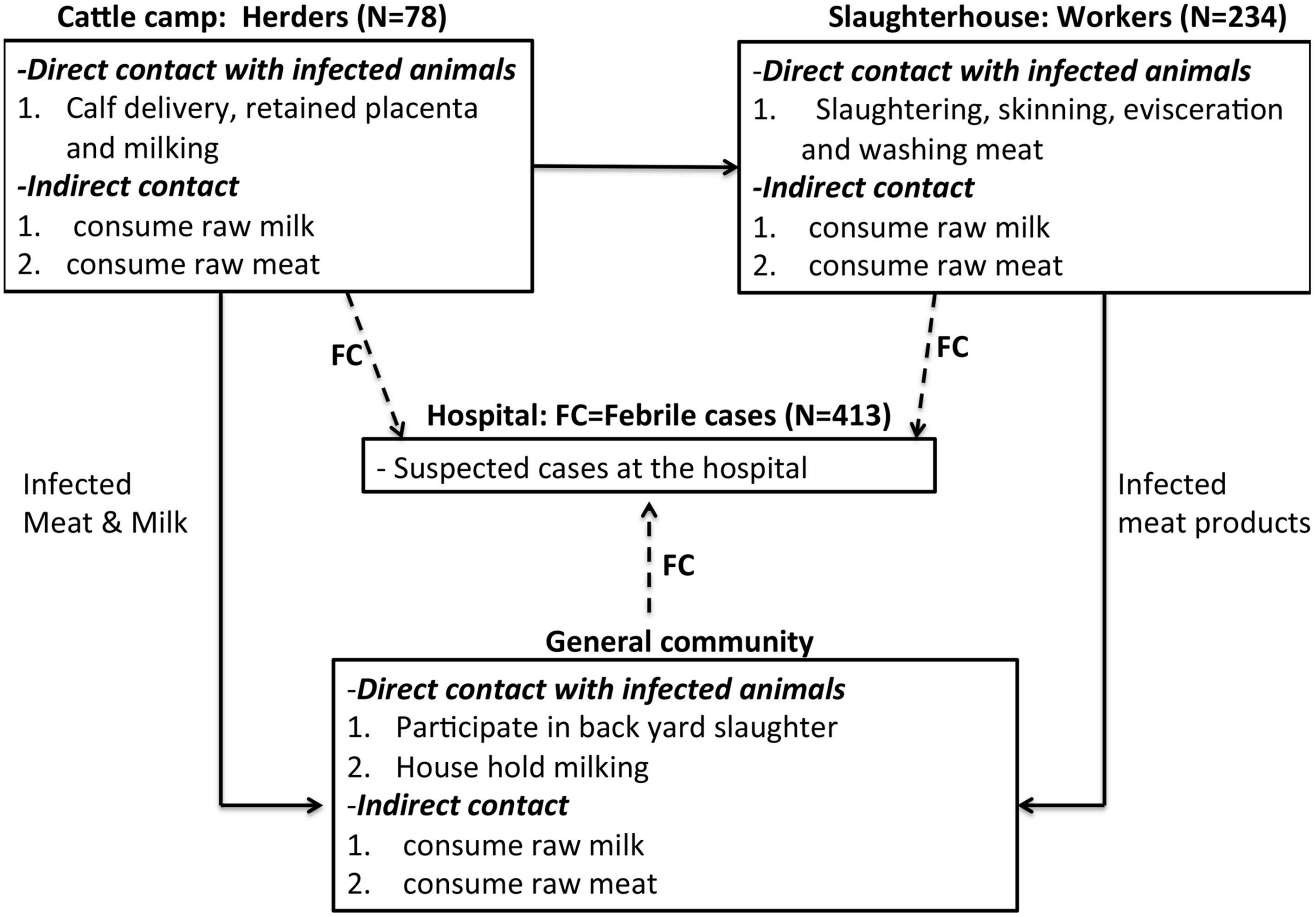
Conceptual Framework showing the risk chain of brucellosis among the three risk groups. FC, febrile cases. Please note that we sampled 87 herders, but nine were dropped from our dataset due to lack of complete data.

This cross-sectional study was carried out among abattoir workers, herders and febrile patients in Bahr el Ghazal region, South Sudan between December 2015 and May 2016. We used both quantitative and qualitative data collection methods to assess the awareness of zoonotic diseases in general but specifically brucellosis; its causes, transmission, and major symptoms of the disease. The study was designed along the brucellosis risk chain including three risk groups in Bahr el Ghazal region; (A) Cattle herders, who owned and reared cattle herds from cattle camps in Tonj and Aweil states. (B) Abattoir workers, these were individuals involved in various cattle slaughter activities in four abattoirs in Tonj, Aweil, Kuajok, and Wau. (C) Febrile cases at Wau hospital, these included all cases received at this hospital with a fever during the period of sampling. The febrile cases originated from Tonj, Aweil, Kuajok, and Wau. The framework in Figure 1 shows the routes of transmission of brucellosis right from the field where cattle were reared, the abattoir where they were slaughtered, and in the community where meat and milk were consumed (see Figure 1). The febrile cases at Wau hospital represent potential brucellosis cases coming from the general community. Note that we focused on four states within Greater Bahr el Ghazal region i.e., Tonj, Aweil, Kuajok, Wau, primarily because of their “relative stability” during a period of armed conflict.

### Study Site

**Figure 2:**
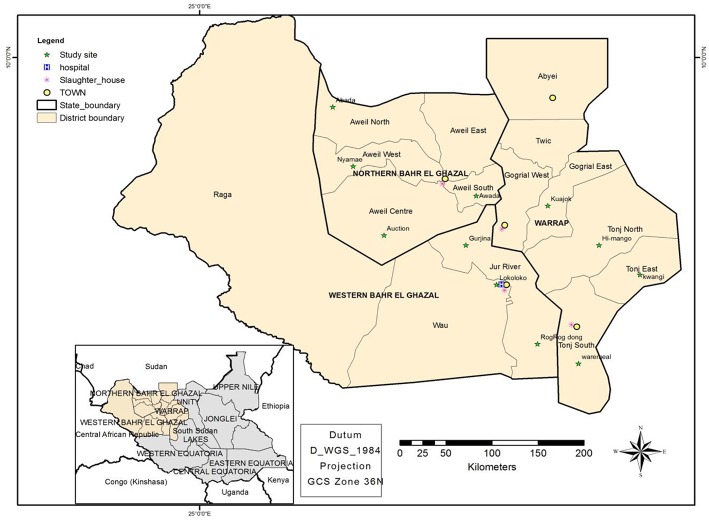
Map showing the study sites, namely; the four states where this study was conducted, the location of abattoirs, cattle camps and Wau-hospital.

Greater Bahr el Ghazal region is located in the Northwestern part of South Sudan. The region consists of vast land with iron plateau and swamps spread within its ten states of Aweil, Aweil East, Gogrial, Lol, Tonj, Twic, Wau, Gok, Eastern Lakes, and Western Lakes (see Figure 2). South Sudan has approximately 12 million heads of cattle, half of which are found in Greater Bahr El Ghazal region (23). Therefore, the majority of the ethnic groups, which occupy these areas, subsist on livestock.

### Sample Size

Our abattoir workers' sample size (*n*) was determined using Epitools online http://epitools.ausvet.com.au based on the following assumptions; a 10% sero-prevalence reported in Omdurman, Sudan (28), a total population of abattoir workers in the four states to be ~ 1000, a desired precision (α) = 0.05, and a detection power (β) = 86% of the c ELISA (29). Then, we had to sample ~ 100 abattoir workers, approximately 25 per abattoir. We however managed to sample *n* = 234 abattoir workers randomly.

Approximately 7500 febrile cases were seen at Wau hospital during the study period. As there were no published brucellosis sero-prevalence estimates for febrile patients in South Sudan, we assumed 50% sero-prevalence. Using the same assumption as in the previous section, we needed to screen 262 febrile cases. However, given that the study was conducted in a time frame longer than initially anticipated with consent, we sampled 416 febrile cases.

Since herds are owned and maintained in cattle camps, this means that large numbers of cattle are clustered in fewer than 150 cattle camps. We conveniently screened 87 herders from 6 cattle camps in two of the four states (Tonj and Aweil). We screened fewer herders than we actually contacted because some did not agree to participate in the study.

### Sampling Strategy, Collection and Transportation

In general, our sampling was purposive in nature, guided by the prevailing security at the time. A list of all abattoir workers from an abattoir in each of the four states was obtained and used to randomly select individuals to screen. From Wau hospital, we conveniently screened febrile cases who presented at Wau hospital during the study period and had consented to be part.

On the other hand, 1–3 cattle camps were selected in each of the two states. We obtained a list of herders in each of the camps, but only took samples from those that consented to participation in this study. From all the participants, a blood sample was drawn from the cephalic vein by our team of registered nurses, approximately 5 ml of blood were collected from each participant. These samples were then kept at room temperature and tilted at an angle of 45° for 6–8 h to allow for clotting. A questionnaire was also administered to each of the participants and here we collected information on the individual's occupation (specific activity within the defined risk groups), age, sex, marital status, ethnicity, knowledge of zoonotic diseases, routine practices and attitudes of the participants were captured through interviews as described in the next section. The sera were aliquoted into new sets of labeled Eppendorf tubes, stored on ice packs and transported to Wau Teaching Hospital Laboratory, and kept in a deep freezer at −80°C. The samples were then transported by air to the Central Diagnostic Laboratory at Makerere University, College of Veterinary Medicine Animal Resources and Biosecurity, Kampala-Uganda.

### Qualitative Data Collection

We conducted eight Focus Group Discussions (FGDs) and Key Informant Interviews (KII). All participants consented to being recorded during the interviews and discussions. Trained note takers were at the same time capturing the whole conversations for cross validation. A trained modulator was involved in engaging the participant (s) in a discussion or interview about brucellosis. This meant that a team of three (3) individuals which consisted of the modulator, person recording and note taker were involved in conducting the qualitative part of our study.

### Focus Group Discussions (FGDs)

Each FGD took up to 1 h of discussion with the participants. The FGD participants included the general population that never participated in the quantitative part of the study. Two Focus groups were held in each of the four states making a total of eight FGDs. The group size was about 6–8 individuals and participants were selected to ensure homogeneity in the group by considering characteristics such as; sex, occupation, owning cattle, location, among other factors.

### Key Informant Interviews (KII)

Key informant interviews were held with veterinary officers, medical professionals and general directors from veterinary and health ministries in the four selected states. Either a medical officer and a veterinary officer or a general veterinary director were interviewed for each state. In this effect, we interviewed two key informants per state, and each KII took approximately half an hour.

### Serology

#### Competitive Enzyme Linked Immune Sorbent Assay (c-ELISA)

A competitive ELISA that was primarily set-up for the detection of anti-*Brucella* antibodies in livestock, was validated for the detection of anti-*Brucella* antibodies in human patients in Tanzania (30). This c-ELISA has been used in both humans and cattle in the same study as that in the Serengeti National Park, Tanzania (31). We have used the same strategy in this study by using the brucellosis Serum P04130-13 and brucellosis antibody test kit from IDEXX (there has been no other test used). The test was performed following the manufacturer's guidelines accessible at www.IDEXX.com.

### Data Management and Analysis

#### Data Assembly

The results from the serology test were then merged with the questionnaire and individual bio data for each risk group in SPSS versions 24. For the purpose of validation, a double-blinded data entry and merge was used, after which the two sets of entries were compared for agreement. The three data sets from each of the risk groups were then merged into one database (SX) which was used for analysis. Qualitative data was transcribed from audio to text, the text was then consolidated with the notes taken during each of the KII and FGDs.

#### Data Analysis

##### Quantitative data

The established dataset was exported to R version 3.4.2. For statistical analysis; descriptive statistics, proportions and percentage of the *Brucella* positive against number of individual variables were run. Univariable logistic regression was used to identify significant bi-variable associations with various individual knowledge and practice variables. Note that at this point, the outcome variable on the individual tests was whether or not an individual tested positive or negative on the c-ELISA. The output was used to select factors that could be used in developing a logistic regression model. This model was developed to identify potential drivers of zoonotic brucellosis, in this area. The model was developed by adding variables in a forward selection process adjusting for confounding, starting with variables that had the lowest *p*-value from the univariable analysis. Only variables that had a *p* < 0.25 were included in the model, these were added and removed to see if they still retained their level of statistical significance (*p* < 0.05), as well as checked for potential confounding effects. The least complex model was chosen based on the lowest Akaike information criterion (AIC). Standard post estimation statistics like the Hosmer-Lemeshow test was also done. The same process was repeated with the outcome variable of (Yes or No) as an evaluation of their awareness that brucellosis is a zoonotic disease. This analysis was aimed at analyzing the factors associated with awareness of which animal diseases (zoonotic diseases) are likely to affect them as well.

### Brucellosis Risk Profiling Using Hierarchical Clustering

This analysis aimed at identifying the age and individual-based occupation activity that represents the highest brucellosis risk within the three groups (herders, Abattoir workers and febrile cases). This was done using the proportion of brucellosis sero-positives cases along the age continuum and specific routine occupational activities. This gives us a measure of exposure risk per occupational activity for a given age. We then generated a metric that represents a measure of this risk;

$$\begin{array}{l} \delta =\frac{\gamma }{\beta } \end{array}$$

**Figure 3:**
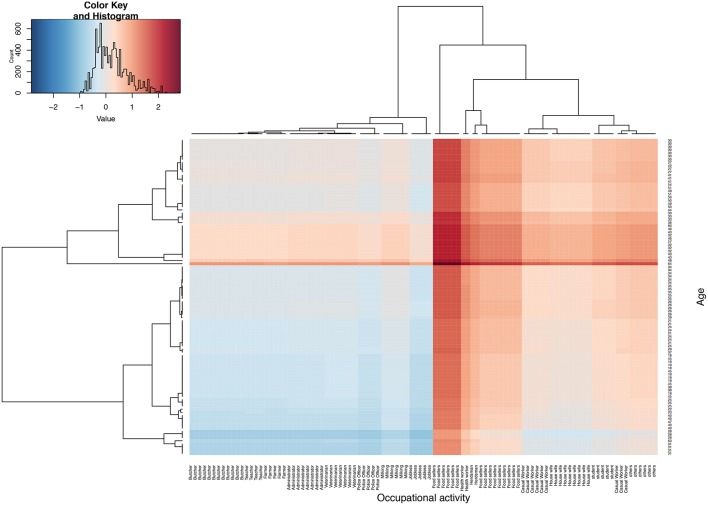
Shows heat map based on parameter δ, four quadrants represents four risk groups. The top right and bottom left are individuals whose risk is highly attributable to their age and occupational activities, respectively. The top left and bottom right quadrant represent groups whose risk could on average be attribute to both age and their occupational activity. The middle band represents a group whose risk remains high across all ages. Note that δ has been log transformed to improve visualization of this parameter.

Where, γ is the proportion of brucellosis sero-positives for a given age, and β is the proportion of brucellosis sero-positives for a given occupational activity. Therefore, metric δ is a ratio of γ and β. When δ is equal or close to 1, then an individual's risk is attributable to both age and their occupational activity. If the δ is >1 then the individual's risk is more attributable to their age while if δ < 1, then risk is more attributable to their occupational activity. The distribution of this metric (δ) is then presented as a color scale from cool to hot, that is to say; blue (lowest) to brown (highest), and plotted as heat map using heat map package in R (Figure 3). Note that in order to improve the clarity of the image and labeling of the heat map, we have used a random subset of the data (*n* = 100).

### Identification of Covariate Patterns for Diagnostic and Public Health Use

This analysis aimed at identifying covariate patterns from a set of explanatory factors that can be used to improve case detection in the existing diagnostic algorithms in Wau hospital, as well as underpin public health interventions. The set of explanatory factors used in this analysis was identified basing on measures of reliability and internal consistency (32) for all the variables in (Supplementary Table 2). We obtained a set of variables with the highest Cronbach's alpha, (factors that were independent but carried equivalent weight). We then created a subset of database with these factors in addition to the column with c-ELISA results (brucellosis status, +/-), this was then transformed using commands in the ELRM package in R to generate a data frame with all the possible combinations of factor levels (covariate patterns), with their corresponding brucellosis status. A binary variable success and failure was generated, success was defined as a positive status for brucellosis and trials as the number of times a unique covariate pattern occurs. The probability of brucellosis for a given covariate pattern was then calculated as; number of success divided by trials. The probability of brucellosis given a covariate pattern ranged from 0 to 1. This minimum and maximum were then used to identify covariate patterns useful for public health interventions and diagnostics, respectively. Note that these were afterwards ranked by the number of trials with the highest number of successes, and probability of brucellosis considered as most predictive.

### Qualitative Data Analysis

Tape-recorded information and notes taken during the FGDs were transcribed and translated from the local language (Arabic) to English, and typed into Microsoft Word (2013). The same was done for the key informant interviews. The analysis of the transcripts into key thematic areas was done as previously described by the Graneheim and Lundman framework (33). The themes included; major aspects regarding zoonotic disease awareness, animal management and contact, occupational practices and health seeking-behavior. The transcripts were independently read by three members of the team to further identify sub themes. The themes as well as sub themes were then broadly mapped to the objectives of the study and used to further explain any identified statistically significant association in the quantitative analysis.

### Ethics Statement

This study involves an administration of questionnaires to the participants, as well as blood sampling from the human participants. For participants younger than 18 years old, consent was obtained from their guardian. Therefore, the study protocol was reviewed and approved by the ethics committee at Ministry of Health of South Sudan (MOH) (S1). We also obtained ethical approval SBLS/REC/15/133 (SBLS.NA.2015 (S2) from the Ethical Review Committee of the College of Veterinary Medicine, Animal Resources and Biosecurity (COVAB), Makerere University, Uganda and Ministry of Agriculture, Animal Industry and Fisheries (MAAIF)—RSS/MLFI/DVS/J/15/7 (S3), South Sudan; Furthermore, informed consent was obtained from the participants before blood collection and questionnaire administration. We also secured the necessary import and export permits to transport samples from South Sudan to Uganda (S4 and S5).

## Results

### Summary Statistics

A total of 725 participants were recruited in this study from three high-risk groups namely; abattoir workers N = 234, febrile cases presenting at Wau outpatient facility *N* = 413, and cattle herders *N* = 78. Majority of the individuals were between 20–40 years of age. The number of males was almost half that of females, majority of who were literate and belonged to the Dinka ethnic group. The individuals in this study participated in a wide variety of occupational-activities as shown in Table 1. The overall zoonotic brucellosis prevalence was estimated at 27.2 % (95% CI = 23.9–30.6) with 32.1% (95% CI = 26.2–38.4), 23.0% (95% CI = 19.1–27.4) and 34.6% (95% CI = 24.4–46.3) for abattoir workers, febrile patients and herders, respectively. Brucellosis prevalence was highest and lowest among the Kerash (42.9 %) as well as the Arabs (11.9 %) ethnic group, respectively (Table 2).

**Table 1:** Univariable and Multivariable logistic regression analysis of socio-demographic factors and zoonotic diseases awareness.

**Factors**	**Level**	**Zoonotic diseases awareness**		**Zoonotic diseases awareness % (95%CI)**	**X^**2**^**	**Un-adjusted *P*-value**	**Adjusted OR (95%CI)**	**Adjusted (*P*-value)**
		**No**	**Yes**					
*Brucella* result	Negative	448	80	15.2(12.3–18.7)	6.94	0.01	1	Ref
	Positive	150	47	23.9(18.2–30.5)	-	-	1.88(1.11–3.18)	0.02^*^
Marital status	Married	401	91	18.5 (15.2–22.3)	0.82	0.37	1	Ref
	Single	197	36	15.5 (11.1–20.8)	-	-	0.97 (0.55–1.72)	0.92
Sex	Female	272	18	6.2(3.8–9.8)	-	-	1	Ref
	Male	326	109	25.1 (21.1–29.4)	41.50	1.18e-10	2.08 (1.06–4.25)	0.04^*^
Occupation	Administration	11	6	35.3(15.3–61.4)	134.26	<2.2e-16	1	Ref
	Butcher	110	62	36.0 (28.9–43.8)	-	-	1.13 (0.36–3.80)	0.84
	Casual worker	10	1	9.1 (4.7–42.8)	-	-	0.60 (0.03–5.24)	0.68
	Farmer^*^	41	0	-	-	-	0.00	0.99
	Health worker	7	3	30.0 (8.1–64.3)	-	-	0.76 (0.12–4.69)	0.77
	Herdsman	6	2	25.0 (4.5–64.4)	-	-	1.02(0.10–8.31)	0.98
	House wife	49	3	5.8 (1.5–16.9)	-	-	0.19(0.003–0.0)	0.05^*^
	Unemployed	30	1	3.2 (1.6–18.5)	-	-	0.11(0.01–0.84)	0.06
	Milker^*^	46	0	-	-	-	0.00	0.99
	Police officer	17	4	19.0 (6.2–52.6)	-	-	0.39 (0.07–1.89)	0.25
	Food sellers	46	10	17.9 (9.3–30.8)	-	-	0.50 (0.13–1.97)	0.31
	Shop keeper	14	3	17.6 (4.6–44.2)	-	-	0.29(0.47–1.56)	0.16
	Student	113	8	6.6 (3.1–13.0)	-	-	0.15(0.04–0.62)	0.01^*^
	Teacher	9	5	35.7 (13.9–64.4)	-	-	0.74(0.14–3.69)	0.71
	Veterinarian	6	14	70.0 (45.7–87.1)	-	-	4.75(1.09–22.8)	0.04^*^
	Self employed	40	2	4.8(8.2–17.4)	-	-	0.12(0.01–0.65)	0.02^*^
	Others	43	3	6.5 (1.6–18.9)	-	-	0.13(0.02–0.64)	0.15
Literacy	Illiterate	247	15	5.7 (3.3–9.4)	38.22	6.3e-10	1	Ref.
	Literate	351	112	24.2 (20.4–28.4)	-	-	2.85 (1.45–5.87)	0.003^**^
Ethnicity	Arab	25	17	40.5 (26.0–56.7)	89.16	<2.2e-16	1	Ref.
	Balanda	80	21	20.8 (13.6–30.2)	-	-	0.58(0.22–1.56)	0.28
	Dinka	351	23	6.1 (4.0–9.2)	-	-	0.15(0.06–0.36)	2.51e05^***^
	Furr	27	21	43.8 (29.7–58.7)	-	-	0.60(0.23–1.53)	0.29
	Jur	37	12	24.5 (13.5–39.2)	-	-	0.67(0.22–1.97)	0.47
	Kerash	28	7	20.0 (9.1–37.5)	-	-	0.56(0.16–1.84)	0.35
	Other	16	7	30.4 (14.1–53.0)	-	-	0.91(0.34–2.42)	0.85
	Zandi	34	19	35.8 (23.4–50.2)	-	-	0.61(0.17–2.17)	0.45

Statistical significance:

*** *<0.001*,

** *0.01*,

* *0.05, AUC = 0.8666, AIC = 511.65. Adjusted and un-adjusted 0R and P value correspond to the multivariable and univariable analysis. Nine herders were dropped from this analysis due to lack of complete data*.

**Table 2:** Univariable and multivariable logistic regression model of socio- demographic factors and zoonotic brucellosis in Bahr el Ghazal.

**Factor**	**Level**	***Brucella*** **results**		**Prevalence % (95%CI)**	**X^**2**^**	**Un-adjusted *P*-value**	**Adjusted OR (95%CI)**	**Adjusted (*P*-value)**
		**Negative**	**Positive**					
Risk group	Abattoir workers	159	75	32.1 (26.2–38.4)	8.63	0.01^*^	-	-
	Febrile cases	318	95	23.0 (19.1–27.4)	-	-	-	-
	Herders	51	27	34.6 (24.4–46.3)	-	-	-	-
Age	0 – 19	99	24	19.5 (13.1–27.8)	6.14	0.05^*^	1	Ref
	20−40	343	130	27.5 (23.5–31.7)	-	-	1.14 (0.65–2.05)	0.65
	41- 80	86	43	33.3 (25.4–42.2)	-	-	1.23 (0.62–2.49)	0.55
Sex	Female	312	123	28.3 (24.1–32.8)	0.54	0.46	1.27 (0.89–1.83)	0.19
	Male	216	74	25.5 (20.6–31.0)	-	-	1	Ref
Marital status	Married	339	153	31.1 (27.1–35.4)	11.31	0.001^**^	1	Ref
	Single	189	44	18.9 (14.2–24.6)	-	-	0.58(0.36–0.91)	0.02^*^
Literacy	Illiterate	180	82	31.3 (25.8–37.3)	3.21	0.07	-	-
	Literate	348	115	24.8 (21.0–29.1)	-	-	-	-
Ethnicity	Arab	37	5	11.9 (4.5–26.4)	11.74	0.11	1	Ref
	Balanda	69	32	31.7 (22.9–41.7)	-	-	3.78 (1.42–12.02)	0.01^*^
	Dinka	274	100	26.7 (22.3–31.5)	-	-	2.48 (1.00–7.51)	0.72
	Furr	34	14	29.2 (17.4–44.3)	-	-	2.82 (0.95–9.55)	0.07
	Jur	39	10	20.4 (10.7–34.7)	-	-	1.91 (0.61–6.72)	0.28
	Kerash	20	15	42.9 (26.7–60.4)	-	-	6.01 (1.97–21.10)	0.003^**^
	Zandi	16	7	30.4 (14.1–53.0)	-	-	3.22 (0.88–12.55)	0.08
	Others	39	14	26.4 (15.6–40.5)	-	-	2.81(0.96–9.51)	0.07
Occupation	Administration	11	6	35.3 (15.2–61.3)	33.81	0.01^*^	-	-
	Butcher	110	62	36.0 (28.9–43.7)	-	-	-	-
	Casual worker	9	2	18.2 (3.2–52.2)	-	-	-	-
	Farmer	26	15	36.6 (22.5–53.0)	-	-	-	-
	Health worker	9	1	10.0 (5.2–45.8)	-	-	-	-
	Herdsman	7	1	12.5 (5.2–45.8)	-	-	-	-
	House wife	40	12	23.1(12.9–37.1)	-	-	-	-
	Unemployed	18	13	41.9 (39.2–74.9)	-	-	-	-
	Milk handlers	31	15	32.6 (19.9–48.1)	-	-	-	-
	Police officer	13	8	38.1 (18.9–61.3)	-	-	-	-
	Food sellers	48	8	14.3 (6.7–26.7)	-	-	-	-
	Shop keeper	16	1	5.9 (3.1–30.7)	-	-	-	-
	Students	97	24	19.8 (13.3–28.2)	-	-	-	-
	Teachers	9	5	35.7 (13.9–64.3)	-	-	-	-
	Veterinarian	13	7	35.0 (16.3–59.1)	-	-	-	-
	Self employed	33	9	21.4 (10.8–37.2)	-	-	-	-
	Others	38	8	17.4 (8.3–31.9)	-	-	-	-

Statistical significance:

** *0.01*,

* *0.05, AUC = 0.63, AIC = 845.32, Nine herders were dropped from this analysis due to lack of complete data*.

### Zoonotic Disease Awareness

In general, we estimate that the Furr 43.8 % (95%CI = 29.7–58.7) and Dinka 6.1 % (95%CI = 4.0–9.2) ethnic groups scored highest and lowest on the assessment for zoonotic diseases (Table 1), respectively. The awareness analysis shows that individuals who were sero-positive for *Brucella* were approximately two times more likely to score highly on the zoonotic diseases awareness assessment than those who were negative (OR = 1.88, 95%CI: 1.11–3.18, *P* = 0.02). So, the infected would have been aware that brucellosis is a zoonotic disease. Females were twice likely to score highly on the zoonotic diseases awareness assessment as compared to the males (OR = 2.08, 95%CI: 1.06–4.25, *P* = 0.04). Among the different occupations, veterinarians were more likely to score highly on the zoonotic disease awareness assessment as compared to administrators (Table 1). Individuals who were literate were approximately three times likely to score highly on the zoonotic disease awareness assessment compared to those who were illiterate (OR = 2.85, 95%CI: 1.45-5.87, *P* = 0.003). It is also important to note that belonging to Dinka ethnic group was associated with a low score on the zoonotic disease awareness assessment (OR = 0.15, 95%CI: 0.06–0.36, *P* = 2.51e-05) when compared to the Arabs Ethnic grouping.

In general, we estimate that up to, 17.5% (95%CI = 14.8–20.5) of the respondents were aware that brucellosis is a zoonotic disease. The brucellosis sero-prevalence among those who were not aware that brucellosis was a zoonotic disease was 25.1% (95%CI = 21.6–28.8), while that among individuals who consumed raw meat and milk was 31.0% (95%CI = 25.2–37.3) and 29.6% (95%CI = 24.3–35.4), respectively (Supplementary Table 1).

### Zoonotic Brucellosis Risk

Table 2 shows the univariable analysis of each socio-demographic explanatory variable and brucellosis status. After adjusting for other variables in the multivariable logistic regression model (Table 2), it was evident that there is a statistically significant relationship between sex, occupation, ethnicity and literacy with zoonotic brucellosis. Individuals who were single were less likely to have brucellosis as compared to those who were married (OR = 0.58, 95%CI: 0.36–0.91, *P* = 0.02), while individuals belonging to the Kerash and Balanda ethnic groups were six and four times more likely to have brucellosis in comparison to those in the Arab ethnic group (OR = 6.01,95%CI: 1.97–21.10, *P* = 0.003), (OR = 3.78,95%CI: 1.42–12.02, *P* = 0.01), respectively.

### Brucellosis Risk Profiling Using Hierarchical Clustering

Figure 3 shows the brucellosis risk profile using hierarchical clustering by age and occupational activities. The figure has four groups; the top right and bottom left quadrants represent groups with the highest risk to zoonotic brucellosis attributable to their age and occupational activity, respectively, that is to say; the top right and bottom left quadrant have the highest and lowest δ, respectively. The bottom left quadrant is the group whose high risk to brucellosis is attributable to their occupational activities, which include; butchers, veterinarian, farmers, milk handlers and teachers predominantly in the early years of their lives (19–40 years). The top right quadrant is the group whose high risk to brucellosis is attributable to their respective age and these include casual workers, housewives and students ranging between 30–50 years of age. On the other hand, the top left and bottom right represent a group of individuals whose δ is almost equal to 1. Therefore, their risk to brucellosis is equally attributable to their age and occupational activities, and they include butchers, veterinarian, farmers, milk handlers and teachers ranging between 30-50, and casual workers, house wives, students ranging between 19–40 years of age. The analysis also highlights a band (group of occupational activities), whose risk is high across all ages and these include health workers, herders and food sellers between 32–57 years of age (Figure 3).

### Covariate Patterns for Clinical Diagnostics and Public Health Interventions

#### Clinical Triage and Diagnostics

The reliability and internal consistence evaluation identified 12 factors used for this analysis (Supplementary Table 3). This analysis identified 422 unique covariate patterns, of which 143 corresponded to a positive brucellosis status. Of these patterns 11 were filtered as having 100% probability for brucellosis with an occurrence (trials) of at least 3 times. There was one pattern that occurred seven times, and on each of these times it was positive for brucellosis (Supplementary Table 3).

#### Public Health Interventions

On the other hand, of the 279-covariate patterns that corresponded to a negative status for brucellosis, 12 patterns were filtered as having 0% probability of brucellosis with the highest number of trials. We identified patterns that occurred 23 and 19 times each, which have 0 probability of brucellosis (Supplementary Table 4). It is noteworthy that the two patterns identified above were observed among females of 20–40 years of age.

### Qualitative Insights on Brucellosis in Bar El Ghazal

#### Awareness of Brucellosis

As part of our FGDs, we wanted to challenge participants on their awareness about zoonotic diseases including brucellosis. We observed that at most, two of the participants in the FGDs in each state were aware that brucellosis is zoonotic. In general, most participants recognized the disease once clinical signs were extensively explained. Once the disease was fully discussed, we got the local names for this condition. This highlighted the fact that they knew the disease, but possibly not the risk it posed to them. There was common agreement that if communities were incentivized by good access to markets for their animals and products, then maybe they would be motivated to get this knowledge about diseases. “*The way out of the national herds is to educate these communities about the correct way of keeping animals and motivate them to adopt selling and buying animals, instead of keeping animals without genuine economic benefit” (FGD in Tonj state)*.

These communities are very patriarchal, and indeed most meetings were almost exclusively males in some states. One of the women challenged the sustainability of any proposed awareness campaigns if it was only to benefit one gender.

“*It is customary that only men are supposed to attend these meetings held in our villages, and from experience, in such meetings is where all educative information is shared, this leaves us the women unaware of some of the key aspects of such diseases.”*

There are basic norms taught to every generation in most of these communities, the norms and practices inadvertently protect our people against infections including zoonotic diseases. Some individuals however believe that there is some level of complacency with in occupational groups “*Most people, farmers or herders actually practice good hygiene and sanitation, and you find that at the end of it all, they have less chances of contracting these zoonotic diseases. Some groups however, like the animal keepers, particularly those with large numbers, they think they know everything about the animals and diseases affecting them. Most of them do not even call local veterinarians to help them when the animal is sick, so that is the kind of environment that can contribute to zoonotic diseases (Key informant in Kuajok State).”*

#### Brucellosis Ranking as a Zoonotic Disease

We wanted to find out how much importance the participants placed on brucellosis. This was after we had discussed the clinical signs with the participants (Local name of the disease in quotes). “*El Homa El Maltiiais” is not a big problem for me, because the cow does not die, and we eat the aborted calf. Diseases like FMD “Albums Al Huma Algulaia,” Anthrax “Alhuma Alfahmia,” East Coast Fever (ECF) “Huma Alwadi Elmutasadea” kill the animal, and they interfere with our movements because we cannot move the animals for long distances and cannot sell animals (FGD in Aweil state)*.

The same question was put to the key informants to gauge their views.

“*We conduct vaccination campaigns annually before the dry season for Black Quarter (BQ), Anthrax*, Contagious Bovine Pleuropneumonia (CBPP) *and* Hemorrhagic septicemia *(HS), however, brucellosis not included,” (DG Wau vet. Hospital*). Participants in a focus group discussion in Wau state also added that; “*Actually for brucellosis vaccination, since we are no longer part of Sudan, our vaccination campaigns are rare, most farmers do it on their own” (FGD in Wau State)*.

#### Veterinary Service Provisions

It is routine for herders to treat their animals without consulting veterinary authority. It appears that veterinarians are seen as state agents, who implement state designed control measures usually with little consultation from the local herders.

“*I have a veterinary pharmacy in the market, and most of the time nomads come to my pharmacy asking for certain drugs without explaining the disease or signs that their animals are suffering from,” (Key informant interview with vet. Officer, Tonj State)*.

#### Perceptions on Occupational Risks

Risk was also associated with “others” and never with the ethnic group or occupational group who were participating in the FGD, as was revealed when veterinary officers were asked on who would likely be at high risk of exposure in their communities. “*People who do not prepare their own food, if you do not prepare your own food you cannot guarantee the hygiene of the environment and products used in this area,” (Key informant interview with vet. Officer, Aweil State)*.

“*Also, there are ethnic groups whose central source of protein is meat, such groups would be exposed more than the average persons, particularly when they eat aborted material,” (Key informant interview with vet. Officer in Kuajok state)*.

The views from focus group discussion with farmers revealed that they believe food sellers, butchers and herders are in more contact with raw meat than anybody else in the community. “*Food sellers normally handle milk and meat, and if they have wounds could they not get this disease? What about nurses and doctors, they touch sick people could they not get exposed as well?” (FGD in Wau, state)*.

## Discussion

Ensuring healthy lives and promoting well-being for all ages is at the heart of the third United Nation's sustainable development goal (SDG) (34). However, the epidemiological complexity of diseases like brucellosis pose a significant challenge to achieving this SDG especially in resource limited settings (35). Availability of robust population-based data is the cornerstone of designing targeted control and elimination strategies. Unfortunately, such data is very rare in resource limited settings like South Sudan (36). Here, we focused on three risk groups; cattle herders, abattoir workers and febrile cases at Wau hospital. We screened individuals for anti-*Brucella* antibodies, examined their awareness of zoonotic diseases, as well as profiled potential socio-anthropological and demographic drivers of the disease. We then use this dataset to identify informative covariate patterns that could be used to improve clinical diagnostics, as well as contextualize public health intervention in South Sudan.

### Zoonotic Brucellosis Prevalence Estimates

The overall brucellosis prevalence estimate in this study was 27.2% (95%CI = 23.9–30.5). This is higher than the prevalence reported in Uganda (17%) (37), Tanzania (5.5%) (21), Ethiopia (4.8%) (38), and Asian countries like Pakistan (21.7%) (39). Because this study takes into account three risk groups in the same geographical region, this represents one of the highest prevalence estimates of zoonotic brucellosis reported on the continent to date (40). Like Pakistan, South Sudan has experienced political and civil unrest since 1950s ~ 68 years which might explain the absence of documentation of the disease dynamics (41). The drivers of the disease in both settings is however likely to be different, given the differences in social structure and practices (42). Indeed, the differences observed in the region between; Uganda, Tanzania, and Ethiopia could be attributable to infrastructure development, awareness and varying animal management systems (43). On the other hand, the rates observed among febrile cases and abattoir workers in our study are significantly lower than what has previously been reported in Libya (44), where a 40% sero-positivity was reported in Yafran municipality. In Libya, the highest sero-prevalence is reported in Jado (47%) and Yifrin (46%) and the prevalence was associated with drinking raw milk among the participants (44). It is noteworthy that in that study, they focused on individuals who spent most time with animals. The authors have also reported a high 31% (95%CI = 28.0–34.2), sero prevalence in cattle in the same areas (45), which emphasized the role played by animals in the epidemiology of brucellosis in such settings.

### A Profile of Zoonotic Brucellosis Risk

Human behavior especially that which overlaps direct interaction with livestock management and processing of animal products is reported to represent significantly higher risk for zoonotic brucellosis (46). The findings in our study characterize the extent to which specific factors affect the risk within such groups. For example, being single appears to be protective for zoonotic brucellosis in comparison to the married individuals. Indeed, such an association has been reported elsewhere (12, 16, 47) which more likely reflects the nature of behavior, contact, or environment of such individuals than their marital status. One can argue that life styles could be at play in this regard, if we assume single individuals are more likely to prepare their own meals. Then the KII informants view on hygienic environment, products and food suggests certainty of hygiene can only be assured at individual level, i.e., one cannot be sure of the food safety unless they prepared it. The model also shows that individuals from specific ethnic groups were more likely to test positive for brucellosis for example; the Kerash and Balanda were six and four times likely to test positive than Arab ethnic grouping. The general lack of granular socio-anthropological data about South Sudan's ethnic groups limits the extent to which some of the observed associations can be explained. However, it can be argued that limited interactions with livestock could influence the general awareness of zoonotic risks by these two ethnic groups. Our data tends to support this notion as we observed that only ~ 25% of the individuals in these two ethnic groups were aware that brucellosis was zoonotic.

Hierarchical clustering identified herders, food sellers and health workers as a group whose risk is high at any given age. Interestingly, although herders and butchers are regularly in contact with animals and their products, the clustering attributes each group's risk to age and occupational activities, respectively. The brucellosis risk profile among butchers has been reported elsewhere (7, 21, 37), where risk was highest among activities that involve direct contact with slaughtering environment (21, 48). We observe that there was a health worker who was sero-positive among the nine tested. It is not clear what the exposure route for this individual was, but it represents a public health problem. The concerns of the communities on such workers are reflected at one of the FGD in Wau state, where participants were asked about certain frontline professions after realizing how brucellosis spreads.

### Zoonotic Disease Awareness

While knowledge is a set of experiences, skills and insights on a specific subject, awareness is the perception and use of this knowledge, therefore, capturing the entirety of awareness for zoonotic diseases is a practically impossible undertaking in such settings. Our study however attempts to capture aspects of this attribute using both qualitative and quantitative data collection techniques. The findings show that female respondents, regardless of risk group were more aware of the zoonotic diseases compared to their male counterparts. This could be because of the gender roles with regards to animal management in this setting, but the finding seems to contradict literature elsewhere (49). Women are usually involved in all domestic activities excluding livestock rearing. This is with the exception of tending to sick cattle left at home, while the apparently healthy ones are taken for grazing by men (50). Moreover, women are excluded from information dissemination activities such as; local meetings, health intervention and planning as was highlighted in one of the FGD in Tonj state.

The observed contradictions could indeed be due to the highlighted exception in routine activities that allows female to observe the basic clinical characteristics of brucellosis, and thus the awareness of its zoonotic potential (51–53). The counter intuitive aspect observed in this study is that individuals who tested positive for brucellosis were twice more likely to be aware that brucellosis is a zoonotic disease. This could be due to complacency among some groups, like the animal keepers particularly those with large numbers (43, 54) as highlighted by some of the key informant interviews.

If we take the above notion, we would then expect individuals from ethnic groups known to keep large herds, such as the Dinka to be the typical example of individuals who test positive and were aware that brucellosis is zoonotic. The data however shows that the Dinka comparatively had lower scores on this assessment which does not fit with the belief. Such contradictions emphasize the complexity in the dynamics behind zoonotic brucellosis (55).

### Improving Clinical Diagnostics and Public Health Interventions

Clinical history, environmental and occupational risks, as well as socio-demographic aspects have for long been used to improve clinical diagnostic algorithms (56). Our covariate analysis reveals three potentially informative patterns from this population. For example, an illiterate 20–40-year female who does not consume raw milk, but washes hands with water or animal urine and experienced no chills, headache, arthritis, fatigue, or hand abrasion resulted into a positive test for all the seven times it was encountered. Similarly, having hand abrasion combined with consumption of raw milk and washing hands with animal urine also resulted into a positive test for all the six times it was encountered. The latter is however only observed among men of the same age bracket. Exploiting such patterns to inform brucellosis diagnostics is critical in such settings where the diagnostic infrastructure is very inadequate. On the other hand, identifying patterns that are consistently associated to a negative test for brucellosis would be useful in guiding public health awareness campaigns. For example; we observe that being literate, not consuming raw milk, not washing hands with animal urine were associated with a negative test to brucellosis in a pattern that occurred twenty-three times. There is utility in considering such approaches when designing and allocating scarce resource for public health interventions. This specific approach is likely to have more utility if used on in risk groups.

### Limitations of the Study

One of the limitations of this study is limited sample size of the herders and the purposive nature of sampling; this inherently introduced some biases in the estimates generated in our statistical analysis. This was unavoidable given the administrative structure of how herds are managed, and the mistrust that exists between herdsmen and government, as well as the tax collection agencies. None-the-less, our modest sample size allowed for more degrees of freedom which in turn improves the robustness of the parameters in generals. It should also be noted that although we expect the general population's dynamics to be reflected by the febrile cases, however, these findings ought to be interpreted in the context of risk groups as defined by this study.

## Conclusion

We report the highest sero-prevalence of zoonotic brucellosis in three risk groups in the East African region. This is compounded by the limited awareness that brucellosis is a zoonotic disease within our study population. We also observe that one among nine (1/9) health workers tested was sero-positive, which represents a genuine public health concern. We identified some social demographic associations with brucellosis, but the qualitative analysis suggests these are more complex and nuanced. Therefore, future studies could benefit from the use of the mixed methods approach to add extensiveness and depth to our understanding of zoonotic disease drivers. Importantly, *Brucella* spp need to be isolated in order to identify the livestock reservoir species. Once livestock reservoir species are identified, intervention strategies, among which vaccination of reservoir livestock species, can be designed and implemented.

## Ethics Statement

This study involves an administration of questionnaires to the participants, as well as blood sampling from the human participants. Therefore, the study protocol was reviewed and approved by the ethics committee at Ministry of Health of South Sudan (MOH) (S1). We also obtained ethical approval SBLS/REC/15/133 (SBLS.NA.2015 (S2) from the Ethical Review Committee of the College of Veterinary Medicine, Animal Resources and Biosecurity (COVAB), Makerere University, Uganda and Ministry of Agriculture, Animal Industry and Fisheries (MAAIF) - RSS/MLFI/DVS/J/15/7 (S3), South Sudan; Furthermore, informed consent was obtained from the participants before blood collection and questionnaire administration. This was also done before the qualitative data acquisition as explained above. We also secured the necessary import and export permits to transport samples from South Sudan to Uganda (S4 and S5).

## Author Contributions

CK, NM, AM, JBM, JG, AJ, and JM: conceptualization. NM, AM, GN, and AJ: data curation. NM, AM, GN, AJ, and JM: formal analysis. NM: investigation. CK, NM, AM, GN, JBM, JG, AJ, and JM: methodology. CK and JM: project administration. CK and AM: resources. AM and JM: software. CK, AM, JBM, JG, and JM: supervision. AM, JM, and CK: validation. AM, JM, and CK: visualization. CK, NM, AM, GN, JBM, JG, AJ, and JM: writing – review and editing.

### Conflict of Interest Statement

The authors declare that the research was conducted in the absence of any commercial or financial relationships that could be construed as a potential conflict of interest.

## References

[1] Refai M. Incidence and control of brucellosis in the Near East region. Vet Microbiol. (2002) 90:81–10. 10.1016/S0378-1135(02)00248-1 12414137

[2] Wyatt HV. Lessons from the history of brucellosis. Rev Sci Tech. (2013) 32:17–25. 10.20506/rst.32.1.2181 23837362

[3] Roth F Zinsstag J Orkhon D Chimed-Ochir G Hutton G Cosivi O. Human health benefits from livestock vaccination for brucellosis: case study. Bull World Health Org. (2003) 81:867–76. 14997239 PMC2572379

[4] The Development of New/Improved brucellosis Vaccines: Report of a WHO Meeting. Geneva: WHO/EMC/ZDI/98 14 (1997).

[5] Report of the WHO Working Group Meeting on brucellosis Diagnosis and Research in Enzyme Immunoassay. Geneva: WHO/CDS/VPH/93.125 (1993).

[6] Young EJ Brucella spp. Principles and Practice of Clinical Bacteriology, 2nd ed. West Sussex: John Wiley & Sons Ltd (2006) p. 265–72 10.1002/9780470017968.ch19

[7] Tsegay A Tuli G Kassa T Kebede N. Seroprevalence and risk factors of brucellosisBrucellosis in small ruminants slaughtered at debre ziet and modjo export abattoirs, Ethiopia. J Infect Dev Countries. (2015) 9:373–80. 10.3855/jidc.4993 25881526

[8] Otte J Grace D Human health risks from the human-animal interface in Asia. Asian Livestock. (2012) 16:121 Available online at: http://www.fao.org/3/i3166e/i3166e00.pdf

[9] Godfroid J Cloeckaert A Liautard JP Kohler S Fretin D Walravens K. From the discovery of the Malta fever's agent to the discovery of a marine mammal reservoir, brucellosis has continuously been a re-emerging zoonosis. Vet Res. (2005) 36:313–26. 10.1051/vetres:2005003 15845228

[10] Memish ZA Balkhy HH. Brucellosis and international travel. J Travel Med. (2004) 11:49–55. 10.2310/7060.2004.13551 14769288

[11] Godfroid J Scholz HC Barbier T Nicolas C Wattiau P Fretin D. Brucellosis at the animal/ecosystem/human interface at the beginning of the 21st century. Prevent Vet Med. (2011) 102:118–131. 10.1016/j.prevetmed.2011.04.007 21571380

[12] World Health Organization The Control of Neglected Zoonotic Diseases: a Route to Poverty Alleviation: Report of a Joint WH. (2006).

[13] Weekly Epidemiological Bulletin, Republic of South Sudan; Integrated Diseases Surveillance and Response. Available online at: http://www.who.int/hac/crises/ssd/south-sudan-epi-update-30april2017.pdf (accessed October 30, 2018).

[14] Crump JA Morrissey AB Nicholson WL Massung RF Stoddard RA Galloway RL. Etiology of severe non-malaria febrile illness in Northern Tanzania: a prospective cohort study. PLoS Negl Trop Dis. (2013) 7:e2324. 10.1371/journal.pntd.0002324 23875053 PMC3715424

[15] McDermott J Grace D Zinsstag J. Economics of brucellosis impact and control in low-income countries. Rev Sci Tech. (2013) 32:249–61. 10.20506/rst.32.1.2197 23837382

[16] Kansiime C Mugisha A Makumbi F Mugisha S Rwego IB Sempa J. Knowledge and perceptions of brucellosis in the pastoral communities adjacent to Lake Mburo National Park, Uganda. BMC Publ Health. (2014) 14:242. 10.1186/1471-2458-14-242 24612845 PMC3975325

[17] Wirth ME Balk D Delamonica E Storeygard A Sacks E Minujin A. Setting the stage for equity-sensitive monitoring of the maternal and child health Millennium development goals. Bull World Health Org. (2006) 84:519–27. 10.2471/BLT.04.019984 16878225 PMC2627391

[18] World Health Organization Working to Overcome the Global Impact of Neglected Tropical Diseases: First WHO Report on Neglected Tropical Diseases. (2010).

[19] Corbel MJ. Brucellosis: an overview. Emerg. Infect. Dis. (1997) 3:213. 10.3201/eid0302.970219 9204307 PMC2627605

[20] Conti LA Rabinowitz PM One health initiative. Infektološki Glasnik. (2011) 31:176–8.

[21] Swai ES Schoonman L. Human brucellosis: seroprevalence and risk factors related to high risk occupational groups in Tanga Municipality, Tanzania. Zoonoses Public Health. (2009) 56:183–7. 10.1111/j.1863-2378.2008.01175.x 18811674

[22] Agasthya A Isloor S Prabhudas K. Brucellosis in high risk group individuals. Indian J Med Microbiol. (2007) 25:28. 10.4103/0255-0857.31058 17377349

[23] Natsios AS Sudan, South Sudan, and Darfur: What Everyone Needs to Know. OUP USA (2012).

[24] Daumerie D Savioli L Working to Overcome the Global Impact of Neglected Tropical Diseases: First WHO Report on Neglected Tropical Diseases. Vol. 1. World Health Organization. (2010).

[25] Osman AE Hassan AN Ali AE Abdoel TH Smits HL Brucella melitensis Biovar 1 and Brucella abortus S19 vaccine strain infections in milk handlers working at cattle farms in the Khartoum Area, Sudan. PLoS ONE. (2015) 10:e0123374 10.1371/journal.pone.0123374 25938483 PMC4418725

[26] Gumaa MM Osman HM Omer MM El Sanousi EM Godfroid J Ahmed AM. Seroprevalence of brucellosis in sheep and isolation of Brucella abortus biovar 6 in Kassala State, Eastern Sudan. Rev Sci Tech. (2014) 33:957–65. 10.20506/rst.33.3.2333 25812219

[27] Godfroid J. Brucellosis in livestock and wildlife: zoonotic diseases without pandemic potential in need of innovative one health approaches. Arch Public Health. (2017) 75:34. 10.1186/s13690-017-0207-7 28904791 PMC5592711

[28] Salman A Elniema A Mustafa E Amona M Hamid A Lmyaa MH Application of different serological tests for the detection of the prevalence of bovine brucellosis in lactating cows in Khartoum State, Sudan. J Appl Ind Sci. (2014) 2:213–8. Available online at: https://www.academia.edu/26660280/Application_of_Different_Serological_Tests_for_the_Detection_of_the_Prevalence_of_Bovine_Brucellosis_in_Lactating_Cows_in_Khartoum_State_Sudan

[29] Bronsvoort BM Koterwas B Land F Handel IG Tucker J Morgan KL. Comparison of a flow assay for brucellosis antibodies with the reference cELISA Test in West African Bos indicus. PLoS ONE. (2009) 4:e5221. 10.1371/journal.pone.0005221 19381332 PMC2667634

[30] John K Fitzpatrick J French N Kazwala R Kambarage D Mfinanga GS. Quantifying risk factors for human brucellosis in rural northern Tanzania. PLoS ONE. (2010) 5:e9968. 10.1371/journal.pone.0009968 20376363 PMC2848606

[31] Shirima GM Kunda JS. Prevalence of brucellosis in the human, livestock and wildlife interface areas of Serengeti National Park, Tanzania. Onderstepoort J Vet Res. (2016) 83:a1032. 10.4102/ojvr.v83i1.1032 27247075 PMC6238685

[32] Gliem JA Gliem RR Calculating, interpreting, and reporting Cronbach's alpha reliability coefficient for Likert-type scales. In: Midwest Research-to-Practice Conference in Adult, Continuing, and Community Education. Columbus, OH: Ohio State University (2003).

[33] Graneheim UH Lundman B. Qualitative content analysis in nursing research: concepts, procedures and measures to achieve trustworthiness. Nurse Educ Today. (2004) 24:105–12. 10.1016/j.nedt.2003.10.001 14769454

[34] Dadzie PS Martin-Yeboah E Tachie-Donkor G Ensuring Healthy Lives and Promoting Well-Being for All: The Role of Ghanaian Academic Libraries in Achieving the 2030 Agenda for Sustainable Development (2016).

[35] Dieckhaus KD Kyebambe PS. Human Brucellosis in Rural Uganda: clinical manifestations, diagnosis, and comorbidities at Kabale regional referral hospital, Kabale, Uganda. Open Forum Infect Dis. (2017) 4:ofx237. 10.1093/ofid/ofx237 29255733 PMC5726460

[36] Musa MT Jahans KL Fadalla ME. Brucella biovars isolated from nomadic cattle in the southern Darfur province of Western Sudan. J Compar Pathol. (1990) 102:49–54. 10.1016/S0021-9975(08)80006-0 2179290

[37] Tumwine G Matovu E Kabasa JD Owiny DO Majalija S. Human brucellosis: sero-prevalence and associated risk factors in agro-pastoral communities of Kiboga District, Central Uganda. BMC Publ Health. (2015) 15:900. 10.1186/s12889-015-2242-z 26374402 PMC4572625

[38] Kassahun J Yimer E Geyid A Abebe P Newayeselassie B Zewdie B. Sero-prevalence of brucellosis in occupationally exposed people in Addis Ababa, Ethiopia. Ethiopian Med J. (2006) 44:245–52. 17447390

[39] Mukhtar F Kokab F. Brucella serology in abattoir workers. J Ayub Med Coll Abbottabad. (2008) 20:57–61. 19610518

[40] Ducrotoy M Bertu WJ Matope G Cadmus S Conde-Álvarez R Gusi AM Brucellosis in Sub-Saharan Africa: current challenges for management, diagnosis and control. Acta Tropica. (2017) 165:179–93. 10.1016/j.actatropica.2015.10.023 26551794

[41] Braha D. Global civil unrest: contagion, self-organization, and prediction. PLoS ONE. (2012) 7:e48596. 10.1371/journal.pone.0048596 23119067 PMC3485346

[42] Harrus S Baneth G. Drivers for the emergence and re-emergence of vector-borne protozoal and bacterial diseases. Int J Parasitol. (2005) 35:1309–18. 10.1016/j.ijpara.2005.06.005 16126213

[43] Yisehak K Gender responsibility in smallholder mixed crop–livestock production systems of Jimma zone, South West Ethiopia. Livestock Res Rural Dev. (2008) 20:12 Available online at: https://www.lrrd.org/lrrd20/1/yise20011.htm

[44] Ahmed MO Elmeshri SE Abuzweda AR Blauo M Abouzeed YM Ibrahim A. Seroprevalence of brucellosis in animals and human populationsin the western mountains region in Libya, December 2006–January 2008. Euro Surveill. (2010) 15:19625–8. Available online at: https://www.eurosurveillance.org/content/10.2807/ese.15.30.19625-en 20684813

[45] Madut NA Muwonge A Nasinyama GW Muma JB Godfroid J Jubara AS. The sero-prevalence of brucellosis in cattle and their herders in Bahr el Ghazal region, South Sudan. PLoS Negl Trop Dis. (2018) 12:e0006456. 10.1371/journal.pntd.0006456 29924843 PMC6010255

[46] Boukary AR Saegerman C Abatih E Fretin D Alambédji Bada R De Deken R. Seroprevalence and potential risk factors for Brucella spp. infection in traditional cattle, sheep and goats reared in urban, periurban and rural areas of Niger. PLoS ONE. (2013) 8:e83175. 10.1371/journal.pone.0083175 24358261 PMC3865157

[47] Earhart K Vafakolov S Yarmohamedova N Michael A Tjaden J Soliman A. Risk factors for brucellosis in Samarqand Oblast, Uzbekistan. Int J Infect Dis. (2009) 13:749–753. 10.1016/j.ijid.2009.02.014 19457689

[48] Ehizibolo DO Ehizibolo PO Ehizibolo EE Sugun MV Idachaba SE The control of neglected zoonotic diseases in Nigeria through animal intervention. Afr J Biomed Res. (2011) 14:81–8. Available online at: https://www.ajol.info/index.php/ajbr/article/view/95237

[49] Amin H Ali T Ahmad M Zafar MI Gender and development: roles of rural women in livestock production in Pakistan. Pak J Agri Sci. (2010) 47:32–36. Available online at: https://www.pakjas.com.pk/papers/9.pdf

[50] Muralidharan K Prakash N Cycling to school: increasing secondary school enrollment for girls in India. Am Econ J. (2017) 9:321–50. 10.1257/app.20160004

[51] Kozukeev TB Ajeilat S Maes E Favorov M. Risk factors for brucellosis–Leylek and Kadamjay districts, Batken Oblast, Kyrgyzstan, January-November, 2003. MMWR Morb Mortal Wkly Rep. (2006) 55(Suppl 1):31–4. 16645580

[52] Bamaiyi P Hassan L Bejo SK Zainalabidin M Seroprevalence of Brucellosis among farmers and veterinary technical staff in Peninsular Malaysia. Sains Malaysiana. (2017) 46:933–43. 10.17576/jsm-2017-4606-13

[53] Abdullah QYM Alkhyat SH Almahbashi AA Al-Nowihi M Al-Thobahni A Al-Bana MNQ Seroprevalence of Brucella Infection among Pregnant Women in Sana'a City, Yemen. J Epidemiol Infect Dis. (2017) 1:00001 Available online at: https://medcraveonline.com/BBIJ/BBIJ-07-00245.pdf

[54] Alhamada AG Habib I Barnes A Robertson I. Risk factors associated with brucella seropositivity in sheep and goats in Duhok Province, Iraq. Vet Sci. (2017) 4:65. 10.3390/vetsci4040065 29215593 PMC5753645

[55] Muleme J Kankya C Ssempebwa JC Mazeri S Muwonge A. A Framework for integrating qualitative and quantitative data in knowledge attitude and practice studies: A case study of pesticide usage in eastern Uganda. Front Publ Health. (2017) 5:318. 10.3389/fpubh.2017.00318 29276703 PMC5727069

[56] Muwonge A Malama S Bronsvoort BM Biffa D Ssengooba W Skjerve E. A comparison of tools used for tuberculosis diagnosis in resource-limited settings: a case study at Mubende referral hospital, Uganda. PLoS ONE. (2014) 9:e100720. 10.1371/journal.pone.0100720 24967713 PMC4072677

