# Supplementary material for: The Epidemiology of Zoonotic Brucellosis in Bahr el Ghazal Region of South Sudan
Source: Front Public Health. 2019 Jun 26;7:156. doi: 10.3389/fpubh.2019.00156 (PMC6607442; doi:10.3389/fpubh.2019.00156)
Supplement: Supplementary file 1 [file Data_Sheet_1.docx]

**SUPPLEMENTARY TABLES**

**Supplementary Table 1:** Univariate analysis of knowledge on zoonotic diseases and risky practices in comparison with the Zoonotic Brucellosis result.

| **Factors** | **Level** | **Total** | **Brucella results** | | **Prevalence (%)** | **X^2^** | **P-value** |
| --- | --- | --- | --- | --- | --- | --- | --- |
|  |  |  | **Negative** | **Positive** |  |  |  |
| Zoonotic diseases  Knowledge | No | 598 | 448 | 150 | 25.1 | 6.94 | 0.01** |
|  | Yes | 127 | 80 | 47 | 37.0 |  |  |
| Consume raw meat | No | 486 | 363 | 123 | 25.3 | 2.31 | 0.13 |
|  | Yes | 239 | 165 | 74 | 31.0 |  |  |
| Consume raw milk | No | 451 | 335 | 116 | 25.7 | 1.08 | 0.30 |
|  | Yes | 274 | 193 | 81 | 29.6 |  |  |
| Wash hands after work | No | 86 | 61 | 25 | 29.1 | 0.09 | 0.77 |
|  | Yes | 639 | 467 | 172 | 26.9 |  |  |
| Wash hands with water | No | 449 | 336 | 113 | 25.2 | 2.14 | 0.14 |
|  | Yes | 276 | 192 | 84 | 30.4 |  |  |
| Wash hands  Water and soap | No | 161 | 119 | 42 | 26.1 | 2.45 | 0.29 |
|  | Some time | 78 | 51 | 27 | 34.6 |  |  |
|  | Yes | 486 | 358 | 128 | 26.3 |  |  |
| Wash hands  With urine | No | 647 | 477 | 170 | 26.3 | 2.04 | 0.15 |
|  | Yes | 78 | 51 | 27 | 34.6 |  |  |

**Supplementary Table 1 shows** Univariate analysis of knowledge on zoonotic diseases and risky practices in comparison with the zoonotic brucellosis result. This table shows how knowledge of brucellosis among respondents as well as their practices affect their brucellosis sero-status. From the table, we can derive assumptions about knowledge and practices of zoonotic brucellosis and their contribution to the respondents’ brucellosis sero-status.

**Supplementary Table 2:** Reliability analysis

| **Factors** | **Raw-alpha**  **95%(CI)** | **Std.alpha** | **G6** |
| --- | --- | --- | --- |
| Risk group | 0.61 (0.59,0.63) | 0.64 | 0.77 |
| Sex | 0.66 (0.64,0.68) | 0.67 | 0.80 |
| Literacy | 0.69 (0.67,0.71) | 0.70 | 0.81 |
| Zoonotic diseases knowledge | 0.68 (0.66,0.70) | 0.70 | 0.81 |
| Do you consume raw milk | 0.66 (0.64,0.68) | 0.67 | 0.80 |
| Wash hands with water | 0.67 (0.65,0.65) | 0.68 | 0.80 |
| Wash hands with water and soap | 0.64 (0.62,0.66) | 0.65 | 0.78 |
| Wash hands with urine | 0.64 (0.62,0.66) | 0.65 | 0.77 |
| Fever | 0.67 (0.65,0.69) | 0.69 | 0.80 |
| Headache | 0.67 (0.65,0.69) | 0.68 | 0.80 |
| Shilling | 0.67 (0.65,0.69) | 0.69 | 0.81 |
| Arthritis | 0.66 (0.64,0.68) | 0.67 | 0.80 |
| Fatigue | 0.67 (0.65,0.69) | 0.68 | 0.80 |
| Brucella results | 0.69 (0.67,0.71) | 0.70 | 0.82 |
| Age-CAT | 0.69 (0.67,0.71) | 0.70 | 0.82 |
| Do you have hands abrasion | 0.66 (0.64,0.68) | 0.68 | 0.80 |

Internal consistency and reliability of the factors included in the model were tested using Cronbach’s alpha as shown in the supplementary table 2. Furthermore, the evaluation for reliability and internal consistency was highest when the standard alpha=0.70, Guttmann’s Lambda 6 ranges from 80-82.

|  |  |
| --- | --- |

**Supplementary Table 3: Covariate patterns identified to aid triage in clinical diagnosis of Brucellosis in Bahr el Ghazal region, South Sudan**

| CP | Sex | Age | Literacy | Raw milk* | Wash* | Wash** | Zoonotic* | Chills | Headache | Arthritis | Fatigue | Abrasion | Brucella | Trails | Success | Prob |
| --- | --- | --- | --- | --- | --- | --- | --- | --- | --- | --- | --- | --- | --- | --- | --- | --- |
| 1 | F | 20-40 | IL | No | Yes | Yes | No | No | No | No | No | No | + | 7 | 7 | 1 |
| 2 | M | 20-40 | IL | Yes | Yes | No | No | No | No | No | No | Yes | + | 6 | 6 | 1 |
| 3 | F | 0-19 | L | No | No | No | No | No | Yes | No | No | No | + | 5 | 5 | 1 |
| 4 | M | 41-80 | IL | No | Yes | Yes | No | Yes | Yes | Yes | Yes | No | + | 5 | 5 | 1 |
| 5 | F | 20-40 | IL | No | Yes | Yes | No | No | No | Yes | Yes | No | + | 4 | 4 | 1 |
| 6 | M | 20-40 | L | Yes | No | No | No | No | No | No | No | Yes | + | 4 | 4 | 1 |
| 7 | F | 0-19 | L | No | No | No | No | No | Yes | No | No | No | + | 3 | 3 | 1 |
| 8 | F | 0-19 | L | No | No | No | No | Yes | Yes | No | No | No | + | 3 | 3 | 1 |
| 9 | M | 20-40 | L | No | No | No | No | No | No | No | No | No | + | 3 | 3 | 1 |
| 10 | M | 20-40 | L | No | No | No | No | No | Yes | No | No | No | + | 3 | 3 | 1 |
| 11 | M | 20-40 | L | Yes | No | No | No | No | No | No | No | No | + | 3 | 3 | 1 |

F= Female, M= Male, L= literate, IL= illiterate, += positive - = Negative, Raw milk*= Consumption of raw milk, Wash*= wash with hands with water, Wash**= Wash hands with urine, Zoonotic* =knowledge of zoonotic diseases, Prob= Probability of Brucellosis, CP covariate patterns

Supplementary table 3 shows the covariate patterns and the corresponding probability of being a brucellosis case in Bahr el Ghazal region. Each row represents a combination of variables for which the outcome is a likely Brucella positive sero-status. Such combinations can be used in clinical diagnosis of brucellosis in resource constrained countries.

**Supplementary Table 4: Covariate patterns identified to aid public health interventions for Brucellosis in Bahr el Ghazal region, South Sudan**

| **CP** | Sex | Age | Literacy | Raw milk* | Wash* | Wash** | Zoonotic* | Chills | Headache | Arthritis | Fatigue | Abrasion | Brucella | Trails | Success | Prob |
| --- | --- | --- | --- | --- | --- | --- | --- | --- | --- | --- | --- | --- | --- | --- | --- | --- |
| 1 | F | 20-40 | L | No | No | No | No | No | Yes | No | No | No | - | 23 | 0 | 0 |
| 2 | F | 20-40 | IL | No | Yes | Yes | No | No | No | No | No | No | - | 19 | 0 | 0 |
| 3 | M | 0-19 | L | No | No | No | No | No | Yes | No | No | No | - | 12 | 0 | 0 |
| 4 | M | 20-40 | IL | Yes | Yes | No | No | No | No | No | No | Yes | - | 9 | 0 | 0 |
| 5 | M | 20-40 | L | No | No | No | No | No | Yes | No | No | No | - | 9 | 0 | 0 |
| 6 | M | 20-40 | L | Yes | No | No | No | No | No | No | No | Yes | - | 9 | 0 | 0 |
| 7 | M | 20-40 | IL | No | Yes | Yes | No | No | No | No | No | No | - | 8 | 0 | 0 |
| 8 | M | 20-40 | IL | Yes | No | No | No | No | No | No | No | Yes | - | 8 | 0 | 0 |
| 9 | M | 20-40 | L | No | No | No | No | No | No | No | No | No | - | 8 | 0 | 0 |
| 10 | M | 20-40 | L | Yes | Yes | No | No | No | No | No | No | Yes | - | 7 | 0 | 0 |
| 11 | M | 41-80 | IL | Yes | Yes | No | No | No | No | No | No | Yes | - | 7 | 0 | 0 |
| 12 | F | 20-40 | L | No | No | No | No | No | No | No | No | No | - | 7 | 0 | 0 |

F= Female, M= Male, L= literate, IL= illiterate, += positive - = Negative, Raw milk*= Consumption of raw milk, Wash*= wash with hands with water, Wash**= Wash hands with urine, Zoonotic* =knowledge of zoonotic diseases, Prob= Probability of Brucellosis, CP covariate patterns

Supplementary table 4 shows the covariate patterns and the corresponding probability of being a non-brucellosis case in Bahr el Ghazal region. Each row represents a combination of variables for which the outcome is negativity for brucellosis. Such combinations can help in saving resources that would have been used in diagnosis of brucellosis. This information can be obtained from history taking from the Hospital and this covariate pattern can then be used as a guide. In addition, health workers can use these covariate patterns to guide public health interventions.
